# Plant Growth Promotion and Biocontrol of Leaf Blight Caused by *Nigrospora sphaerica* on Passion Fruit by Endophytic *Bacillus subtilis* Strain GUCC4

**DOI:** 10.3390/jof9020132

**Published:** 2023-01-18

**Authors:** Junrong Wang, Shun Qin, Ruidong Fan, Qiang Peng, Xiaojing Hu, Liu Yang, Zengliang Liu, Ivan Baccelli, Quirico Migheli, Gabriele Berg, Xiaoyulong Chen, Tomislav Cernava

**Affiliations:** 1 College of Agriculture, College of Tobacco Science, Guizhou University, Guiyang 550025, China; 2 International Jointed Institute of Plant Microbial Ecology and Resource Management in Guizhou University, Ministry of Agriculture, China Association of Agricultural Science Societies, Guizhou University, Guiyang 550025, China; 3 Guizhou-Europe Environmental Biotechnology and Agricultural Informatics Oversea Innovation Center in Guizhou University, Guizhou Provincial Science and Technology Department, Guiyang 550025, China; 4 College of Ecology and Environment, Tibet University, Lhasa 850012, China; 5 Guangxi Crop Genetic Improvement Biotechnology Laboratory, Nanning 530007, China; 6 Microbiology Research Institute, Guangxi Agricultural Science Academy, Nanning 530007, China; 7 Institute for Sustainable Plant Protection, National Research Council of Italy (CNR), 50019 Sesto Fiorentino, Italy; 8 Dipartimento di Agraria and NRD–Nucleo di Ricerca sulla Desertificazione, Università degli Studi di Sassari, 07100 Sassari, Italy; 9 Institute of Environmental Biotechnology, Graz University of Technology, 8010 Graz, Austria

**Keywords:** passion fruit, endophytic *Bacillus*, enzyme activity, antagonisms, plant growth promotion

## Abstract

Passion fruit (*Passiflora edulis* Sims) is widely cultivated in tropic and sub-tropic regions for the production of fruit, flowers, cosmetics, and for pharmacological applications. Its high economic, nutritional, and medical values elicit the market demand, and the growing areas are rapidly increasing. Leaf blight caused by *Nigrospora sphaerica* is a new and emerging disease of passion fruit in Guizhou, in southwest China, where the unique karst mountainous landscape and climate conditions are considered potential areas of expansion for passion fruit production. *Bacillus* species are the most common biocontrol and plant-growth-promotion bacteria (PGPB) resources in agricultural systems. However, little is known about the endophytic existence of *Bacillu*s spp. in the passion fruit phyllosphere as well as their potential as biocontrol agents and PGPB. In this study, 44 endophytic strains were isolated from 15 healthy passion fruit leaves, obtained from Guangxi province, China. Through purification and molecular identification, 42 of the isolates were ascribed to *Bacillus* species. Their inhibitory activity against *N. sphaerica* was tested in vitro. Eleven endophytic *Bacillus* spp. strains inhibited the pathogen by >65%. All of them produced biocontrol- and plant-growth-promotion-related metabolites, including indole-3-acetic acid (IAA), protease, cellulase, phosphatase, and solubilized phosphate. Furthermore, the plant growth promotion traits of the above 11 endophytic *Bacillus* strains were tested on passion fruit seedlings. One isolate, coded *B. subtilis* GUCC4, significantly increased passion fruit stem diameter, plant height, leaf length, leaf surface, fresh weight, and dry weight. In addition, *B. subtilis* GUCC4 reduced the proline content, which indicated its potential to positively regulate passion fruit biochemical properties and resulted in plant growth promotion effects. Finally, the biocontrol efficiencies of *B. subtilis* GUCC4 against *N. sphaerica* were determined in vivo under greenhouse conditions. Similarly to the fungicide mancozeb and to a commercial *B. subtilis*-based biofungicide, *B. subtilis* GUCC4 significantly reduced disease severity. These results suggest that *B. subtilis* GUCC4 has great potential as a biological control agent and as PGPB on passion fruit.

## 1. Introduction

Agricultural sustainability has been jeopardized in the last few decades by the massive use of agrochemicals. In major crops, productivity losses due to different kinds of plant diseases range from 21% to 31% worldwide [1]. In addition, many plant pathogens have developed resistance to different chemical pesticides [2]. Consequently, it has become difficult to control certain plant diseases due to the paucity of efficient compounds [3]. Moreover, consecutive farming practices and increasing food demand have aggravated these issues [4]. Many pesticides have difficulty decomposing into simple and less-hazardous elements. As a result, toxic residues may persist in the environment, with adverse effects on human health [5]. By the year of 2050, the world population is projected to reach 9.3 billion people. The rapid increasing demand for food and nutrition requires new solutions about plant disease management [6,7]. A growing awareness of environmental safety and human health due to the use of synthetic chemicals [8] has prompted sustainable management practices with less reliance on chemical pesticides. In recent years, various disease management strategies were adopted to reduce yield losses and mitigate disease emergencies [9]. For example, the use of host resistance genes is regarded as a promising means. However, managing diseases based on a single gene has proven ineffective due to the evolution of subpopulations overcoming resistance traits [10]. Therefore, it is important to explore and develop sustainable, environmentally friendly, and efficient plant disease management approaches, as well as new resources. Biological control, including the utilization of endophytes as biocontrol agents against phytopathogens and as elicitors of plant growth promotion (PGP) to reduce the application of chemicals, has become an ideal substitute for synthetic agrochemicals.

Passion fruit (*Passiflora edulis* Sims) is an important plant of the Passifloraceae family, widely grown in tropical and subtropical regions for the production of fruits, cosmetics, and pharmacological products [11]. Its high economic, nutritional, and medicinal values have led to a rapid increase in market demand over the past decade [12]. In China, it is mainly produced in southern regions, such as Hainan, Fujian, Guangxi and Guangdong provinces [7]. Guizhou, a unique karst landscape area on the Yunnan–Guizhou plateau, is considered a potential production area for passion fruit. However, due to the lack of local varieties and relatively low temperatures, plant pathogens are frequently reported to affect passion fruit production [7,13]. Leaf blight caused by *N. sphaerica* is a new and emerging disease on passion fruit grown in Guizhou [14]. Recently, *Nigrospora* spp., including *N. sphaerica* have been reported as causal agents for leaf blight disease over a broad spectrum of hosts (e.g., fruits, vegetables, and oilseeds) in East Asia, with incidence rates ranging from 2% to 90%, exhibiting significant strain–host specificity [15,16,17]. For passion fruit leaf blight caused by *N. sphaerica* WYR007, the disease incidence was up to 70% [14]. The disease occurred on both young and old leaves, starting from the margins and then extending to the entire leaves. The color of the affected tissue was brown with a yellow halo in the early period and then gradually turned to gray. The disease could cause serious economic losses to local growers. To date, sustainable management strategies, including potential biocontrol resources, remain largely unexplored. In addition, little is known about the presence, biocontrol, and PGP potential of *Bacillus* spp. in the endophytic habitat of passion fruit.

Endophytes are groups of bacteria and fungi that colonize plant tissues as part of their life cycle and do not show any apparent pathogenic effect [18,19]. They are associated with almost all tissues of the host plant, including the intercellular spaces of the cell walls, the vascular bundles, and the reproductive organs, such as flowers, fruits, and seeds [20]. In addition, the existence of endophytes in sterile regenerating tissues of micropropagated plants indicate that soil is not their exclusive habitat [10]. Plants provide major nutrients and protective niches for endophytes, while endophytic microbes produce useful metabolites and systemic signals [21,22]. Environmental factors including soil type, nutrients, and biotic/abiotic stresses affect such interrelationships and lead to a high diversity in the endophytic community in various ecological niches, as well as to the different abilities of endophytes to assist plant growth and health [23,24,25]. Endophytic bacteria are known for their ability to promote plant growth directly or indirectly through a variety of metabolic activities. They could facilitate the acquisition of mineral resources, such as phosphorus, potassium, zinc and iron, and regulate the production of plant hormones, such as gibberellin and cytokinin [26]. Moreover, they may directly suppress the growth of phytopathogens by antagonistic activities, such as the colonization of the ecological niches, nutritional competition, and the induction of host systemic resistance [27]. Hence, by utilizing one or more these mechanisms, endophytic bacteria may positively influence plant growth and health, improve productivity, and reduce disease incidence. Among endophytic bacteria, *Bacillus* spp. are one of the most effective and promising groups that have already been studied and applied in agriculture. Many *Bacillus* strains were developed as plant-growth-promotion bacteria (PGPB) and biofungicide against plant diseases [28,29,30]. Early products were developed from rhizospheric and epiphytic *Bacillus* strains. *Bacillus* L324-92R displays bioactivity against three root diseases of wheat *(Rhizoctonia* root rot, *Pythium* root rot, and *Gaeumannomyces graminis* var. *tritici*) [31]; *B. subtilis* BSn5 has antibacterial activity against bacterial pathogens [31]. Aravind et al. [32] reported the anti-nematode activity of endophytic bacteria *B. megaterium* BP17 against plant-pathogenic burrowing nematodes (*Radopholus similis*). In recent years, endophytic *Bacillus* strains have also been developed as new biofertilizers and biofungicide. Yield Shield (Bayer CropScience Inc., USA) is a commercial product consisting of endophytic *B. pumilus* GB34 (*B. pumilus* INR7), which is designed to exploit the ability of the strain to induce systemic resistance (ISR) and PGP [33]. Another product, Bio-Yield (Bar Biologics Inc., USA), is a combination of *B. subtilis* GB122 (*B. subtilis* GB03) and endogenous *B. amyloliquefaciens* GB99 (*B. amyloliquefaciens* IN937a) [34,35], whose purpose is mainly to accelerate the growth and development of plants. In some cases, endophytic *Bacillus* spp. showed both higher PGP activity and antagonism than those of rhizospheric and epiphytic strains [36]. Moreover, up to now, most of the products were designed to be applied to the root system. In the plant phyllosphere, wind, rains, and other environmental factors could influence the colonization and persistence of beneficial microbes [37,38]. Therefore, endophytic PGPB in the phyllosphere are more promising to control server or new leaf diseases and display higher potential to be developed as excellent biofertilizers and biopeptides.

The aim of this study was to identify endophytic bacteria displaying beneficial traits to passion fruit. *Bacillus* spp. were isolated from healthy passion fruit leaves in the open field. Upon molecular characterization, we aimed to assess their potential as biocontrol agents against *N. sphaerica* and PGP on passion fruit. The specific objectives of the study were to (1) screen the antagonisms of endophytic *Bacillus* strains against *N. sphaerica* in a double culture assay; (2) screen their production of PGP-related metabolites and enzymes in vitro, including IAA, proteases, cellulases, and their phospholytic abilities; and (3) evaluate the PGP activity of promising strains and the control effect of *B. subtilis* GUCC4 against *N. sphaerica* in comparison with the synthetic fungicide mancozeb and *B. subtilis* NCD_2 from a commercial biofungicide under greenhouse conditions.

## 2. Materials and Methods

### 2.1. Sample Collection

The samples were collected in May, 2021, in Fulu Village, Santang Township, Nanning District, Guangxi, China (22°56′4″ N, 108°24′1″ E). Fifteen healthy passion fruit leaves were collected from five passion fruit plants in different locations of the sampling site. Each leaf was collected with sterile tweezers and gloves and placed in a separate sterile plastic bag to avoid contamination. Afterwards, all the samples were stored on ice and in separate cooling boxes until arrival in the Microbiology Laboratory (International Jointed Institute of Plant Microbial Ecology and Resource Management in Guizhou University, Ministry of Agriculture, China Association of Agricultural Science Societies, Guiyang, China) for further studies.

### 2.2. Isolation of Endophytic Bacillus spp.

Each leaf was processed separately and cut into small pieces with a sterile blade. Approximately 0.5 g of leaf tissues was obtained from each leaf sample. Leaves were gently washed with sterile distilled water to remove dust particles. For surface sterilization, each leaf sample was shaken for 30 s in a sterile flask containing 75% *v*/*v* ethanol and then placed in a sterile flask containing 4% *w*/*v* (NaClO) sodium hypochlorite solution for 3 min. To remove any remaining NaClO, they were rinsed three times with sterile distilled water (dH_2_O) for 5 min. Then, they were washed in washing solution, and 1/10 of the total volume was plated on nutrient agar (NA) medium to verify the absence of contaminants. Subsequently, the leaf tissues were finely homogenized with sterile pestle and mortar in 3 mL dH_2_O and left to macerate for 30 min, and the suspension was plated in serial dilutions on NA medium. Each serial dilution was prepared in triplicate, and the plates were incubated at 28 °C. After 5 days, morphologically distinct colonies were picked up and purified in Luria–Bertani (LB) agar plates. The morphology of each isolate was examined microscopically. Bacteria-like isolates were grown in LB broth medium for 24 h at 28 °C. Sterile glycerol was then added to the bacterial culture to a final concentration of 30%, and the bacterial–glycerol suspension was stored at −80 °C until further analysis.

### 2.3. DNA Extraction, Amplification, and Sequencing

The endophytic isolates were grown in LB broth medium for 24 h at 28 °C. Genomic DNA was exacted using MicroElute Genomic DNA Kit (Omega Bio-Tek Co., Ltd., Norcross, GA, USA) according to the manufacturer’s protocol. Subsequently, PCR amplifications were conducted with the primer 27F (5′-AGAGTTGATCCTGGCTCAG-3′) and 1492R (5′-GTTACCTTGTTACGACTT-3′) for the 16S rRNA gene [39] and the primer UP1 (5′-GAAGTCATCATGACCGTTCTGCAYGCNG GNGGNAARTTYGA-3′) and UP1r (5′-AGCAGGGTACGGATGTGCGAGCCRTCNACRTCNG CRTCNGTCAT-3′) for the *gyr*B gene [40]. 

Each 25 µL PCR reaction contained 12.5 µL of Taq mixture, 1 µL of each primer, 1 µL of genomic DNA template, and 9.5 µL double-distilled water (ddH_2_O). The PCR cycling conditions for 16S rRNA gene were 30 cycles of denaturation at 94 °C for 30 s, annealing at 56 °C for 30 s, extension at 72 °C for 90 s, and a final extension at 72 °C for 10 min. The PCR cycling conditions for *gyr*B gene were 35 cycles of denaturation at 94 °C for 30 s, annealing at 55 °C for 45 s, extension at 72 °C for 1 min, and a final extension at 72 °C for 10 min. The PCR products were then sequenced at Sangon Biotech (Shanghai, China). The sequences of the two genes from all of the isolates were compared with public databases using NCBI BLASTN online (http://www.ncbi.nlm.nih.gov/ accessed on 1 March 2022). Furthermore, phylogenetic trees based on the 16S rRNA gene and the *gyr*B gene were constructed by using the maximum likelihood method in MEGA 6.0. 

### 2.4. In Vitro Antagonism against N. Sphaerica

The isolate *N. sphaerica* WYR007 from our previous study [14] was used in the experiments. The antagonistic activity of *Bacillus* spp. strains against *N. sphaerica* was determined by dual culture assay on potato dextrose agar (PDA) plates. Briefly, an agar–mycelium plug (5 mm diameter), obtained from the edge of an actively growing colony of *N. sphaerica* was placed in the center of each PDA plate. Then, in each diagonal direction, 5 µL of *Bacillus* cell suspension (1 × 10^6^ CFU/mL) was inoculated at 20 mm distance from the center of plate. Afterwards, plates were incubated for 72 h at 28 °C. Only plates inoculated with *N. sphaerica* were used as a control. The antagonistic activity was determined by calculating the percentage of the growth inhibition of *N. sphaerica* compared to the control, according to the following formula: Inhibition rate (%) = [(control colony diameter − treated colony diameter)/(control colony diameter)] × 100 [41]. The experiment was repeated twice in three replicates.

To verify the antagonistic activity of volatile compounds produced by *Bacillus* spp. strains against *N. sphaerica*, the partition-plate technique was used [42]. *Bacillus* strains were challenged with *N. sphaerica* on partition plates, which enables the movement of volatiles alone without any direct contact between the microbes. The pathogen inoculated alone into the partition plate was maintained as control and incubated at 28 °C for 7 days. Afterwards, the percent inhibition of *N. sphaerica* was calculated. The experiment was repeated twice in three plates.

The antagonistic activity of *Bacillus* spp. fermentation broths against *N. sphaerica* was determined. Each *Bacillus* strain was incubated with constant shaking at 200 rpm in LB broth at 30 °C for 48 h. After incubation, the fermentation broth was centrifuged at 10,000 rpm for 5 min. The supernatant was then filtered through a 0.22 µm polycarbonate membrane to remove any cellular debris. In each PDA plate, an agar–mycelium plug (5 mm diameter) of *N. sphaerica* was placed in the center of the PDA plate containing 2 mL sterile supernatant of the *Bacillus* fermentation broth. *N. sphaerica* placed sterile supernatant plate containing no *Bacillus* fermentation broth PDA plate were used as a control. The inhibitory activity was measured after 5 days of incubation at 28 °C. The experiment was repeated twice in three plates.

### 2.5. In Vitro Screening of Secondary Metabolites

Indole-3-acetic acid (IAA) synthesis: IAA production by the *Bacillus* strains was determined as described previously [43]. Briefly, 5 μL of *Bacillus* cell suspension (1 × 10^6^ CFU/mL) was incubated with constant shaking at 180 rpm in 5 mL LB broth amended with 100 mg/L tryptophan (Sangon Biotech (Shanghai) Co., Ltd., Shanghai, China) in the dark at 30 °C for 48 h. Five mL of the liquid culture was centrifuged for 10 min at 10,000 rpm. Two mL of the supernatant was mixed with 100 μL of 10 mM orthophosphoric acid and 4 mL of Salkowski reagent (1 mL of 0.5 M FeCl_3_ in 50 mL of 35% HClO_4_). The tubes were incubated at room temperature for 25 min. The development of a pink color indicated IAA production, which was quantified spectrophotometrically at 530 nm. The concentration of IAA in the culture was determined by linear regression analysis using a calibration curve of pure IAA (y = 0.0121x − 0.0257, R^2^ = 0.9685) as the standard. The experiment was repeated twice in three replicates.

Phosphate solubilization: The phosphate-solubilizing activity of *Bacillus* strains was assessed using a plate assay in Pikovaskaya’s medium [44], which contains insoluble tricalcium phosphate as the sole phosphate source. Five microliters of *Bacillus* cell suspension (1 × 10^6^ CFU/mL) was pipetted in the center of a Pikovaskaya’s medium dish and incubated at 28 °C for 7 days. Phosphate dissolution was determined by the presence or absence of a clear zone of hydrolysis below the colony on the agar plate. Experiments were repeated twice in triplicate.

Protease production: Protease production ability of the *Bacillus* strains was determined according to Xu et al. [44] with minor modifications. Briefly, 5 μL of *Bacillus* cell suspension (1 × 10^6^ CFU/mL) was spotted on a skim milk agar (SMA) medium plate. The protease production ability was qualitatively evaluated by the presence of a transparent zone around the *Bacillus* colony after 7 days of incubation at 28 °C. The experiment was repeated twice in three replicates.

Amylase production: Modified from Marten et al. [45], the amylase production ability of the *Bacillus* strains was determined on soluble starch agar medium (10 g peptone, 5 g yeast extract, 2 g soluble starch, and 20 g agar in 1 L distilled water, pH 7.0). Five microliters of *Bacillus* cell suspension (1 × 10^6^ CFU/mL) was inoculated in the center of a soluble starch agar plate and incubated at 28 °C for 2 days. The ability to hydrolyze amylase was qualitatively evaluated by the appearance of a halo zone around the colonies. The experiment was repeated twice in three replicates. 

Cellulase production: The cellulase production ability of the *Bacillus* strains was determined on carboxymethyl cellulose (CMC) agar (10 g peptone, 10 g yeast extract, 10 g CMC, 5 g NaCl, 1 g KH_2_PO_4_, and 20 g agar in 1 L distilled water, pH 7.0) medium, containing 0.2% (*w*/*v*) Congo red [45]. After 5 days of incubation at 28 °C, the ability of isolates to hydrolyze cellulose was determined by the appearance of a clear zone around the colonies. The experiment was repeated twice in three replicates.

### 2.6. Plant Growth Promotion Traits in the Greenhouse 

Passion fruit (cv. Panama Red) were used to test the plant growth promotion traits. The pot experiment was conducted in plastic pots with 24.5 cm × 26.6 cm height and diameter in the month of May–August, 2021, with an average temperature (25 ± 4 °C) in the departmental greenhouse, College of Agriculture, Guizhou University, Guiyang, China. Healthy passion fruit seedlings (plant height 50 cm) in the vegetative growth stage were transplanted to 150 g of peat-based soil matrix containing perlite (Hunan Xianghui Agricultural Technology Development Co., Ltd., Yueyang, China).

Eleven *Bacillus* strains were screened from the previous in vitro studies and used in the pot experiment. They could produce a variety of PGP-related secondary metabolites, as well as showing >65% inhibitory activity against *N. sphaerica*. They were grown in LB broth at 30 °C with constant shaking at 180 rpm for 48 h. Afterwards, the cell suspension of each strain was collected and adjusted to 1 × 10^6^ CFU/mL for further experiments. 

In each treatment (represented by single PGPB strains), 12 passion fruit seedlings were inoculated with the *Bacillus* cell suspension. On each seedling, 50 mL of suspension was evenly sprayed on the front and back of all leaves of the plant. The same number of seedlings were established as the control group, in which equal amounts of sterile water were evenly sprayed on passion fruit leaves. The inoculations were performed at 10-day intervals. The plant height, stem width, maximum leaf length, fresh weight, dry weight, chlorophyll content, and the activity of peroxidase (POD), superoxide dismutase (SOD), catalase (CAT), ascorbate peroxidase (APX), malondialdehyde (MDA), and proline (Pro) in the passion fruit leaves were measured 30 days after the first inoculation. Plant-biomass-related measurements followed previously described methods [46]. The soil plant analysis development (SPAD) values of chlorophyll were determined by the SPAD-502 method [47]. SOD, CAT, POD, APX, MDA, and Pro were determined according to the method of Wang et al. [48]. 

### 2.7. Biological Control Traits against N. Sphaerica in the Greenhouse 

The best performing strain in the PGP traits, *B. subtilis* GUCC4, was selected to verify its biocontrol efficiency against *N. sphaerica* in greenhouse compared with the fungicide mancozeb (Dow AgroSciences, Zionsville, IN, USA), which is reported to be active against *N. sphaerica* [49]. Additionally, a commercial biofungicide based on *B. subtilis* NCD_2 (Tech Green Biochemical Technology Co., Ltd., Hongkong, China) was used as the reference strain. Passion fruit (cv. Panama Red) seedlings of the same growth stage (8–12 leaves) were transferred to plastic plant pots (11 cm × 12.7 cm) containing peat-based sterilized substrate (pH 5.5–7.0, “Xiangnongzhengke”, Hunan Xianghui Agricultural Technology Development Co., Ltd., China). All plants were watered with tap water twice a week until the end of the experiment. Disease incidence was calculated as the percent of diseased leaves over all the leaves. Conidial suspensions of *N. sphaerica* WYR007 (prepared from 1-month-old colonies in 0.05% Tween 20 buffer and adjusted to a concentration of 1 × 10^3^ conidia/mL) were sprayed onto passion fruit leaves (200 μL per leaf) one week after transplantation. On the same day, cell suspensions of *B. subtilis* GUCC4 (1 × 10^6^ CFU/mL) and *B. subtilis* NCD_2 (1 × 10^6^ CFU/mL) were sprayed on passion fruit leaves (5 mL per leaf), respectively. After 14 days, an equal amount of *B. subtilis* cell suspension was applied. In the fungicide control treatment, mancozeb was dissolved in sterile water (adjusted to a concentration of 2.5 mg/mL, active ingredients of pesticides accounting for 43%) and then applied to passion fruit leaves (5 mL per leaf). Passion fruit seedlings inoculated only with *N. sphaerica* WYR007 were use as the inoculated-control. Seedlings treated with tap water were used as the non-inoculated control. Each treatment had 17 seedlings. The disease incidence on passion fruit leaves was determined 28 days after transplanting, calculated as the average percentage of diseased leaves among all leaves in each plant.

### 2.8. Statistical Analysis

All experimental data were expressed as mean ± standard deviation. Analysis of variance was completed using the Statistical Package for the Social Sciences (SPSS V.11; SPSS Inc., Chicago, IL, USA). The one-way ANOVA followed by post hoc analysis was used to compare mean values among treatments at the 5% level of significance (*p* = 0.05).

## 3. Results

### 3.1. Identification of Endophytic Bacteria and Screening of Their Antagonisms against N. sphaerica

**Figure 1:**
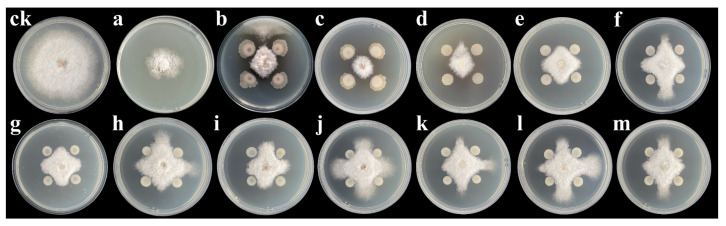
Inhibition of *N. sphaerica* by 11 *Bacillus* strains in comparisons to the control, synthetic fungicide mancozeb, and reference strain *B. subtilis* NCD_2. Treatments, (**ck**): control, (**a**): mancozeb, (**b**): *B. subtilis* NCD_2, (**c**): *B. subtilis* GUCC8, (**d**): *B. subtilis* GUCC4, (**e**): *B. cereus* GUCC7, (**f**): *B. cereus* GUCC3, (**g**): *B. cereus* GUCC6, (**h**): *B. cereus* GUCC9, (**i**): *B. cereus* GUCC11, (**j**): *B. cereus* GUCC2, (**k**): *B. cereus* GUCC5, (**l**): *B. cereus* GUCC1001, (**m**): *B. cereus* GUCC10.

In total, 42 strains of endophytic *Bacillus*-like bacteria were isolated from 15 passion fruit leaves. Based on DNA extraction and the PCR amplification and sequencing of the 16S rRNA gene, 27 strains were identified as *B. cereus*, 3 strains were *B. anthracis*, 2 strains were *B. subtilis*, 2 strains were *B. altitudinis*, 2 strains were *B. wiedmanni*, 2 strains were *B. thuringiensis*, 1 strain was *B. pumilus*, 3 strains were *Bacillus* sp., and 2 strains were *Agrobacterium tumefaciens* (Table 1). In a dual-culture assay of the 42 *Bacillus* strains against *N. sphaerica*, the antagonistic activities, evaluated as the inhibition rate, ranged from 0.00% to 75.35% (Table 1). Among them, 11 strains showed inhibition rates that were above 65%. They were GUCC8 (*B. subtilis*), GUCC4 (*B. subtilis*), GUCC9 (*B. cereus*), GUCC7 (*B. cereus*), GUCC1001 (*B. cereus*), GUCC11 (*B. cereus*), GUCC6 (*B. cereus*), GUCC2 (*B. cereus*), GUCC5 (*B. cereus*) GUCC10 (*B. cereus*), and GUCC3 (*B. cereus*) (Figure 1), and their inhibitory activities were calculated as: 75.35%, 71.16%, 69.21%, 69.84%, 69.46%, 68.82%, 67.55%, 68.74%, 68.99%, 69.04%, and 69.53%, respectively. 

### 3.2. Phylogenetic Analysis of Potential Bacillus Strains 

**Figure 2:**
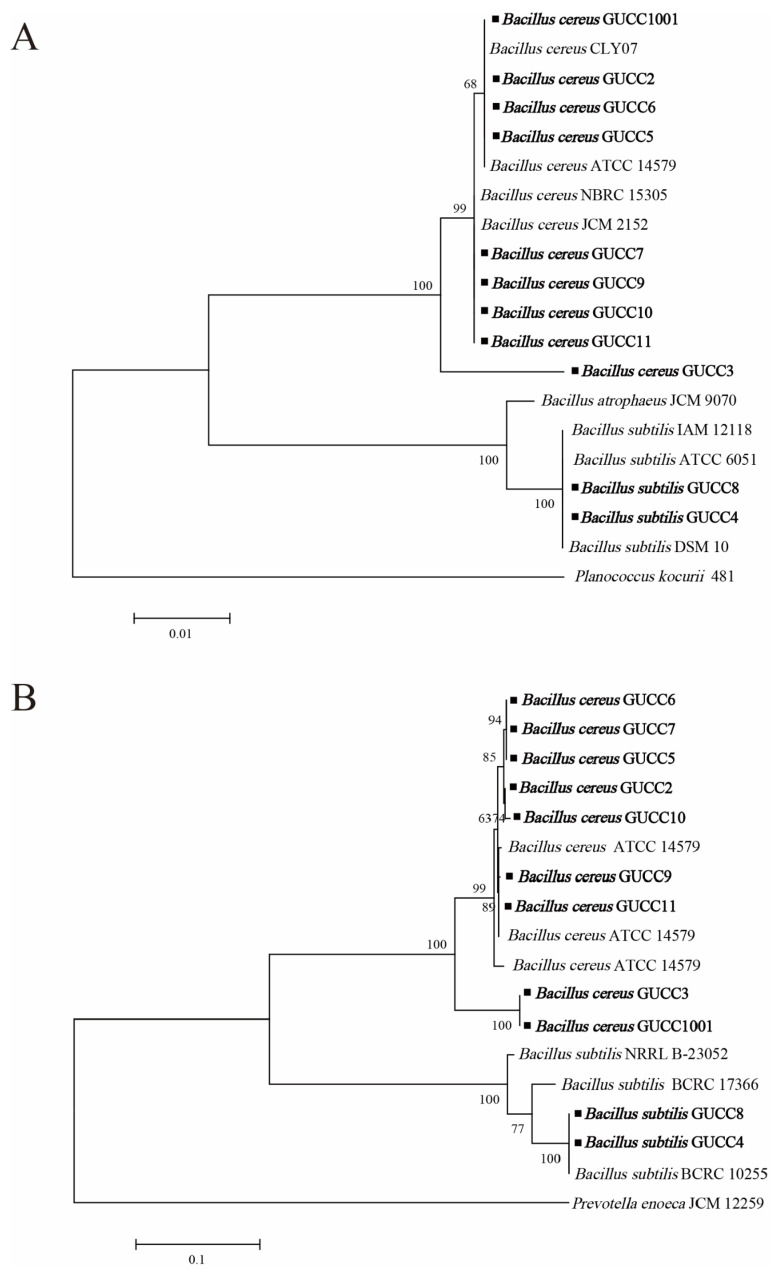
Phylogenetic tree based on 16S rRNA (**A**) gene and *gyr*B (**B**) gene sequences. The strains from this study were highlighted in bold.

In addition to the 16S rRNA gene sequences of GUCC1001, GUCC2, GUCC3, GUCC4, GUCC5, GUCC6, GUCC7, GUCC8, GUCC9, GUCC10, and GUCC11 (Table 1), the PCR amplification products of their *gyr*B genes were sequenced, and the sequences were submitted to the GenBank database (accession numbers: ON908211, ON908201, ON908202, ON908203, ON908204, ON908205, ON908206, ON908207, ON908208, ON908209, and ON908210, respectively). Phylogenetic analysis of the 11 potential Bacillus strains was conducted, and phylogenetic trees were constructed based on the 16S rRNA gene and *gyr*B gene sequences, respectively (Figure 2). Compared with sequences of the type strains, the results confirmed that the strains GUCC1001, GUCC2, GUCC3, GUCC5, GUCC6, GUCC7, GUCC9, GUCC10, and GUCC11 belong to the *B. cereus* group. Simultaneously, strain GUCC4 and strain GUCC8 are ascribed to the *B. subtilis* group.

### 3.3. Inhibitory Activity of Volatile Compounds and Culture Filtrates from Bacillus Strains against N. sphaerica

**Figure 3:**
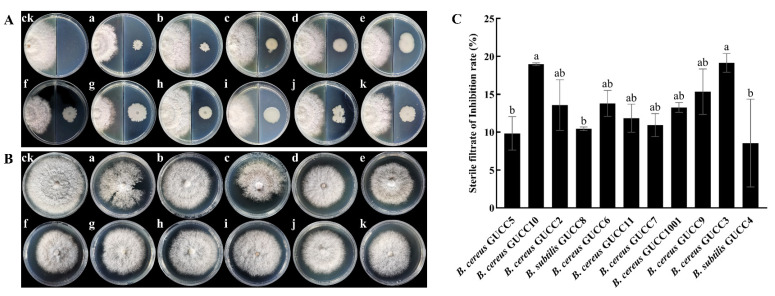
(**A**). Inhibition of *N. sphaerica* by volatile compounds of 11 Bacillus strains treatments, ck: control, a: *B. subtilis* GUCC8, b: *B. subtilis* GUCC4, c: *B. cereus* GUCC7, d: *B. cereus* GUCC3, e: *B. cereus* GUCC6, f: *B. cereus* GUCC9, g: *B. cereus* GUCC11, h: *B. cereus* GUCC2, i: *B. cereus* GUCC5, j: *B. cereus* GUCC1001, k: *B. cereus* GUCC10. (**B**). Inhibition of *N. sphaerica* by culture filtrate of 11 Bacillus strains treatments, ck: control, a: *B. subtilis* GUCC8, b: *B. subtilis* GUCC4, c: *B. cereus* GUCC7, d: *B. cereus* GUCC3, e: *B. cereus* GUCC6, f: *B. cereus* GUCC9, g: *B. cereus* GUCC11, h: *B. cereus* GUCC2, i: *B. cereus* GUCC5, j: *B. cereus* GUCC1001, k: *B. cereus* GUCC10. (**C**). Inhibition of *N. sphaerica* by culture filtrate of 11 Bacillus strains. Data in the table are presented as the means ± standard deviation. Different letters in the same column indicate statistical significance (*p* < 0.05).

The volatile compounds released by GUCC8 (*B. subtilis*), GUCC7 (*B. cereus*), GUCC5 (*B. cereus*), GUCC10 (*B. cereus*), GUCC2 (*B. cereus*), GUCC11 (*B. cereus*), and GUCC6 (*B. cereus*) showed an inhibitory effect on *N. sphaerica* (Figure 3A). Moreover, the culture filtrates of all the 11 potential antagonistic strains showed the inhibition of the growth of *N. sphaerica* hyphae (Figure 3B). The inhibitory effect ranged from 8.55% to 19.14% (Figure 3C). 

### 3.4. In Vitro Screening of Secondary Metabolites

As shown in Table 2, except for *B. cereus* GUCC7, all of the potential strains synthesized IAA in the range from 2.278 to 5.044 μg/mL. At the same time, all of the 11 strains produced amylase, protease, and cellulase. In addition, only *B. subtilis* GUCC4 and *B. subtilis* GUCC8 showed phosphate solubilization activity.

### 3.5. Plant Growth Promoting Effect of Endophytic Bacillus Strains in Greenhouse 

Under greenhouse conditions, *B. cereus* GUCC3 and *B. subtilis* GUCC4 significantly increased all the biomass components of passion fruit, including plant height, stem width, leaf length, leaf surface area, fresh weight, and dry weight (Table 3). In contrast, *B. cereus* GUCC6 did not show any significant effect on the growth promotion of passion fruit. In addition, the other eight strains showed strain–biomass component-specific effects. For instance, *B. cereus* GUCC8 significantly increased the plant height and stem width, as well as leaf length and leaf surface area. However, it could not increase the fresh and dry weights of the passion fruit.

**Figure 4:**
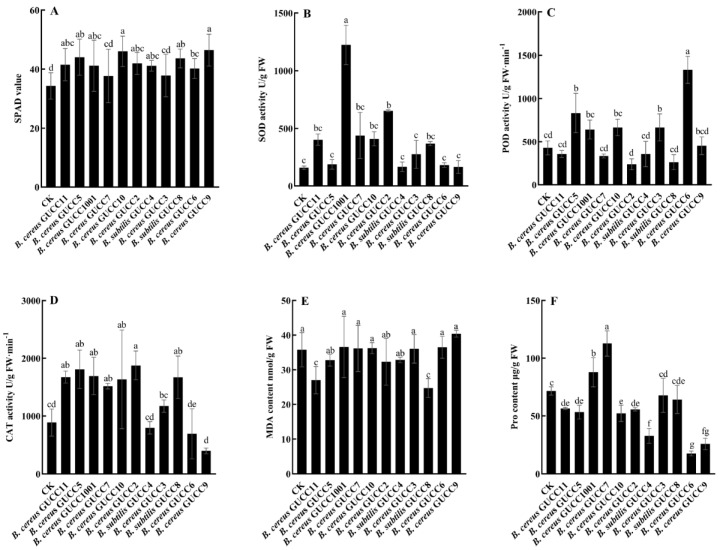
The effects of different Bacillus strains on total chlorophyll content (**A**), SOD (**B**), POD (**C**), CAT (**D**) enzyme activities, total MDA (**E**), and Pro content (**F**) in passion fruit leaves. All data represents the means ± standard deviation for three biological replicates. Values with different letters in the same column indicate statistical significance (*p* < 0.05).

The effects of 11 potential strains on the physiological and biochemical properties were extremely strain-specific (Figure 4). There was no single strain that could positively influence all the determined properties, including chlorophyll content (SPAD values); activities of superoxide dismutase (SOD), peroxidase (POD), catalase (CAT); and contents of ascorbate peroxidase malondialdehyde (MDA) and proline (Pro). In detail, the application of *B. subtilis* GUCC4, *B. cereus* GUCC11, *B. cereus* GUCC5, *B. cereus* GUCC1001, *B. cereus* GUCC10, *B. cereus* GUCC2, *B. cereus* GUCC6, *B. cereus* GUCC9, and *B. subtilis* GUCC8, significantly increased the chlorophyll content (SPAD values) of the passion fruit leaves. *B. cereus* GUCC1001 and *B. cereus* GUCC2 significantly increased the SOD activities, and *B. cereus* GUCC5 and *B. cereus* GUCC3 significantly increased the POD activities. Moreover, *B. cereus* GUCC11, *B. cereus* GUCC5, *B. cereus* GUCC1001, *B. cereus* GUCC7, *B. cereus* GUCC10, *B. cereus* GUCC2, and *B. subtilis* GUCC8 significantly increased the CAT activities. In addition, *B. cereus* GUCC11 and *B. subtilis* GUCC8 could significantly reduce the MDA contents. Concurrently, *B. cereus* GUCC11, *B. cereus* GUCC5, *B. cereus* GUCC10, *B. cereus* GUCC2, *B. cereus* GUCC6, *B. cereus* GUCC9, and *B. subtilis* GUCC4 could significantly reduce the Pro contents.

### 3.6. B. subtilis GUCC4 Biological Control of Leaf Blight in Greenhouse Experiment

The disease incidences on passion fruit leaves were determined 28 days after transplanting. The disease incidence was 42.76%, 37.39%, and 52.72% in seedlings treated with *B. subtilis* GUCC4, mancozeb, and *B. subtilis* NCD_2, respectively, while it was 75.45% in the inoculated control (Table 4). Compared to the inoculated control, *B. subtilis* GUCC4, mancozeb, and *B. subtilis* NCD_2 showed 43.33%, 50.44%, and 30.12% protection, respectively (Table 4).

## 4. Discussion

Passion fruit is an important woody plant due to its high economical, nutritional, and medicinal values [50]. In the last decade, its market demand has consistently increased worldwide, including in China [12]. However, the cultivation and production of passion fruit, especially in low-temperature regions, including mountainous landscapes, is challenged by biotic damage, such as pests and phytopathogens [14]. Meanwhile, abiotic stresses, such as coldness and nutrient limitation, may negatively affect their growth [51,52]. Endophytic bacteria, including *Bacillus* spp., could directly or indirectly promote plant growth through various metabolic activities, stimulate host defense by inducing systemic resistance, and directly suppress or compete with the pathogens [26,27]. In general, *Nigrosopra* spp. are mainly considered plant endophytes [53]. Recently, *N. sphaerica* was reported to be the causal agent of leaf blight disease on different plants [15,17], including passion fruit [14]. To date, sustainable management strategies, including potential biological control resources, remain largely unexplored for controlling plant leaf blight caused by *N. sphaerica*. Furthermore, little is known about the existence, biocontrol, and PGP potential of *Bacillus* spp. in the endophytic habitats of passion fruit.

In this study, we identified a selection of endophytic *Bacillus* spp. beneficial to the host in healthy passion fruit leaves. Eleven isolates from healthy passion fruit leaves displayed effective in vitro antagonism against *N. sphaerica* strain WYR007, the causal agent of passion fruit leaf blight. Sequence homology analysis of 16S rRNA gene allowed the identification of the strains. The isolates GUCC2, GUCC3, GUCC5, GUCC6, GUCC7, GUCC9, GUCC10, GUCC11, and GUCC1001 were identified as *B. cereus*, and GUCC4 and GUCC8 were identified as *B. subtilis*. The results were confirmed by the sequence homology analysis of the *gyr*B gene. In addition, we found that volatile of isolates *B. subtilis* GUCC8, *B. cereus B. cereus* GUCC7, *B. cereus* GUCC5, *B. cereus* GUCC10, *B. cereus* GUCC2, *B. cereus* GUCC11, and *B. cereus* GUCC6 could inhibit the growth of *N. sphaerica* mycelia. These findings were consistent with previous studies that *Bacillus* species could release volatile organic compounds with antifungal properties, including benzene compounds, aromatic hydrocarbons, ketones, aldehydes, alkyl groups, sulfides, pyrazines, and alcohols [43,54,55,56].

To further investigate the PGP and biocontrol potential of these endophytic strains, we determined their IAA production capacity, phosphate solubilization capacity, and various enzymatic activities (protease, cellulase, amylase). All of them could produce protease, cellulase, and amylase. Except for *B. cereus* GUCC7, other strains could synthesize IAA. However, only *B. subtilis* GUCC4 and *B. subtilis* GUCC8 showed phosphate solubilization activity. Vassilev et al. [57] reported that the solubilization of insoluble phosphate by microbial activity usually induces the secretion of certain metabolites, mainly iron carriers, lytic enzymes, and phytohormones, which are involved in the suppression of plant pathogens. It was found that iron carrier production and phosphate solubilization were involved in the growth-promoting activity of antagonistic *Pseudomonas aeruginosa* Rh323 [58]. Moreover, phosphate solubilization accompanied by the production of IAA may contribute to the growth-promoting activity of *P. aeruginosa* BRp3 [59]. Gandhi et al. [60] reported that rice inter-root associates of *Chryseobacterium aquaticum* PUPC1 produced antifungal protease, displaying inhibitory effect on mycelial growth, spore germination, and the nucleation of phytopathogenic fungi. Previous studies suggested that the starch hydrolysis ability of *B. subtilis* could assist host plants in utilizing complex carbon sources and enhancing resistance to biotic stresses including phytopathogens [46]. 

Furthermore, we evaluated the PGP activity of these promising strains under greenhouse conditions and found that *B. cereus* GUCC3 and *B. subtilis* GUCC4 significantly increased all of the biomass components of passion fruit, including plant height, stem width, leaf length, leaf surface area, fresh weight, and dry weight. Our findings were similar to those in previous studies, e.g., Hashem et al. [61] reported that *B. subtilis* BERA71 has a plant-growth-promoting effect (in terms of root length, stem diameter, fresh and dry weight). Similarly, *B. cereus* strains isolated from maize and eucalyptus also promoted the growth and development of maize and eucalyptus when they acted on plants [62,63]. However, due to the fact that *B. cereus* is currently considered a potential human pathogen [64,65,66], *B. cereus* GUCC3 was excluded in our study for further in vivo biocontrol deficiency evaluation.

PGPB from *Bacillus* group have been extensively studied for their double role in biological control against phytopathogens [67,68]. They generally promote plant growth by triggering the production of auxins, glycosides, and other metabolites, enhancing plant vegetative capacity, as well as protecting the plants from both biotic and abiotic stresses through various mechanisms [69]. We determined the biocontrol efficiency of endophytic *B. subtilis* GUCC4 to control *N. sphaerica* in the greenhouse compared with the synthetic fungicide mancozeb, which was reported to be active against *N. sphaerica* [49]. Additionally, *B. subtilis* NCD_2 from a commercial biofungicide was used as reference strain. The disease incidence was 75.45% in the inoculated control. Both *B. subtilis* GUCC4, mancozeb, and *B. subtilis* NCD_2 demonstrated significant effects in the reduction of disease incidence. Their showed 43.33%, 50.44%, and 30.12% protection, respectively. These findings are in accordance with previous reports that *B. subtilis* strains could significantly reduce the leaf disease incidence in different plants [70,71,72], including leaf blight caused by pathogenic fungi [73,74]. Interestingly, there were no significant differences between *B. subtilis* GUCC4, mancozeb, and *B. subtilis* NCD_2. In other words, our strain showed similar and comparable performance to both the commercial fungicide and the biofungicide. This was different with other *B. subtilis* strains showing significantly lower efficiency compared with synthetic fungicides against the same pathogen on the same hosts [75,76,77]. Therefore, *B. subtilis* GUCC4, in addition to its significant effects in promoting passion fruit seedling growth, has the potential to be further applied for the management of passion fruit leaf blight. Further field studies should be conducted to evaluate its potential to be developed as both biofungicides and biofertilizer, particularly for passion fruit production.

Moreover, detailed informations on the interactions between endophyte and passion fruit are desirable: the precise PGP and biocontrol mechanisms on passion fruit, as well as the effects on phyllosphere microbiome, the ability to induce disease/stress resistant genes, and the production of novel secondary metabolites should be further elucidated.

## Figures and Tables

**Table 1:** Identification of endophytic bacterial isolates from passion fruit leaves based on the comparison of 16S rRNA sequences with the type strains in the database and their inhibition rates of *N. sphaerica*.

Endophytic Bacterial Isolate	Accession Number in NCBI Database (Number of Nucleotide)	Closely Related Type Strain	Tentative Endophytic Bacterial Designation	Sequence Similarity in NCBI (%)	Inhibition Rate (%)
GUCC1001	OM004035	CLY07	*B. cereus*	100	69.463 ± 0.012 a
GUCC2	ON882051	CLY07	*B. cereus*	100	68.743 ± 0.002 a
GUCC3	ON882052	JCM 2152	*B. cereus*	100	69.527 ± 0.001 a
GUCC4	ON882053	ATCC 6051	*B. subtilis*	100	71.163 ± 0.000 a
GUCC5	ON882054	ATCC14579	*B. cereus*	100	68.988 ± 0.002 a
GUCC6	ON882055	ATCC14579	*B. cereus*	100	67.550 ± 0.003 a
GUCC7	ON882056	JCM 2152	*B. cereus*	100	69.847 ± 0.007 a
GUCC8	ON882057	ATCC 6051	*B. subtilis*	100	75.353 ± 0.011 a
GUCC9	ON882058	JCM 2152	*B. cereus*	100	69.210 ± 0.002 a
GUCC10	ON882059	JCM 2152	*B. cereus*	100	69.043 ± 0.001 a
GUCC11	ON882060	JCM 2152	*B. cereus*	100	68.823 ± 0.003 a
GUCC1011	OM319531	Gvt-Sh-12	*B. cereus*	100	27.437 ± 0.040 e
GUCC1012	OM319532	ATCC 14579T.112	*B. cereus*	100	48.450 ± 0.030 b
GUCC1013	OM319533	1910ICU267	*B. altitudinis*	100	-
GUCC1014	OM319534	HYSJ134	*B. anthracis*	100	49.103 ± 0.037 b
GUCC1015	OM319535	MSM-S1	*Bacillus* sp.	95.84	-
GUCC1016	OM319536	TS1	*B. cereus*	99.93	31.550 ± 0.061 cde
GUCC1017	OM319537	HYSJ134	*B. anthracis*	100	49.017 ± 0.037 b
GUCC1018	OM319538	MLS-3-7	*A. tumefaciens*	100	-
GUCC1019	OM319539	ATCC 14579T.112	*B. cereus*	100	-
GUCC1020	OM319540	NA161	*B. cereus*	100	37.707 ± 0.038 bcde
GUCC1021	OM319541	ER6	*B. wiedmannii*	100	47.507 ± 0.045 bc
GUCC1022	OM319542	NS26	*B. cereus*	100	-
GUCC1023	OM319543	HYSJ134	*B. anthracis*	100	-
GUCC1024	OM319544	XS 24-5	*B. cereus*	100	-
GUCC1025	OM319545	D51	*Bacillus* sp.	100	-
GUCC1026	OM319546	2	*B. thuringiensis*	100	50.390 ± 0.212 b
GUCC1027	OM319547	NS25	*B. cereus*	100	25.537 ± 0.041 e
GUCC1028	OM319548	LXJ11	*B. cereus*	100	-
GUCC1029	OM319549	MLS-1-10	*A. tumefaciens*	100	-
GUCC1030	OM319550	XS 6-4	*B. cereus*	100	-
GUCC1031	OM319551	DGT10	*Bacillus* sp.	100	-
GUCC1032	OM319552	4589	*B. cereus*	100	30.023 ± 0.035 de
GUCC1033	OM319553	K44	*B. cereus*	100	47.070 ± 0.033 bc
GUCC1034	OM319554	MP2B-4	*B. cereus*	100	-
GUCC1035	OM319555	2_T22	*B. thuringiensis*	100	25.897 ± 0.047 e
GUCC1036	OM319556	AM3	*B. cereus*	100	41.840 ± 0.037 bcde
GUCC1037	OM319557	FJAT-45863	*B. cereus*	99.93	-
GUCC1038	OM319558	41KF2bT.26	*B. altitudinis*	100	-
GUCC1039	OM319559	Gvt-Sh-12	*B. cereus*	100	-
GUCC1040	OM319560	PB4	*B. pumilus*	100	25.537 ± 0.041 e
GUCC1041	OM319561	EH20	*B. wiedmannii*	100	49.140 ± 0.036 b
HUCC1042	OM319562	LXJ74	*B. cereus*	100	25.537 ± 0.041 e
GUCC1043	OM319563	XS 24-5	*B. cereus*	100	45.430 ± 0.063 bcd

Note: Data are presented as the means ± standard deviation. Different letters in the same column indicate statistical significance (*p* < 0.05).

**Table 2:** In vitro screening of secondary metabolites produced by endophytic *Bacillius* spp. from this study.

Strain	Amylase	Protease	Cellulase	Phosphate Solubilization	IAA(μg/mL)
*B. cereus* GUCC5	+	+	+	−	3.171 ± 0.073 b
*B. cereus* GUCC10	+	+	+	−	3.612 ± 0.048 b
*B. cereus* GUCC2	+	+	+	−	3.281 ± 0.048 b
*B. subtilis* GUCC8	+	+	+	+	5.044 ± 0.443 a
*B. cereus* GUCC6	+	+	+	−	3.446 ± 0.048 b
*B. cereus* GUCC11	+	+	+	−	2.278 ± 0.089 c
*B. cereus* GUCC7	+	+	+	−	−
*B. cereus* GUCC1001	+	+	+	−	3.198 ± 0.048 b
*B. cereus* GUCC9	+	+	+	−	3.364 ± 0.126 b
*B. cereus* GUCC3	+	+	+	−	3.198 ± 0.095 b
*B. subtilis* GUCC4	+	+	+	+	3.529 ± 0.048 b

Note: +: Capable of secreting this enzyme. −: No ability to secrete this enzyme. Data in the table are presented as the means ± standard deviation. Different letters in the same column indicate statistical significance (*p* < 0.05).

**Table 3:** The effects of endophytic Bacillus strains on growth parameters of passion fruit seedlings.

Treatment	Stems Width (mm)	Plant Height (cm)	Leaf Length (cm)	Fresh Weight (g)	Leaf Surface Area (cm^2^)	Dry Weight (g)
Control (CK)	2.972 ± 0.179 d	2.333 ± 0.882 g	9.522 ± 0.140 e	1.551 ± 0.067 e	84.116 ± 3.629 de	0.453 ± 0.040 d
*B. cereus* GUCC11	3.678 ± 0.119 bc	10.167 ± 1.092 f	10.222 ± 0.361 de	1.855 ± 0.154 de	72.617 ± 6.018 ef	0.501 ± 0.008 bcd
*B. cereus* GUCC5	4.223 ± 0.209 a	13.833 ± 1.481 de	11.089 ± 0.439 bcd	2.517 ± 0.169 ab	89.859 ± 6.044 de	0.567 ± 0.026 ab
*B. cereus* GUCC1001	3.593 ± 0.148 bc	21.667 ± 3.283 bc	10.533 ± 0.282 cde	1.802 ± 0.106 de	101.768 ± 2.356 bcd	0.467 ± 0.013 cd
*B. cereus* GUCC7	3.613 ± 0.129 bc	14.667 ± 1.856 de	10.422 ± 0.171 cde	1.791 ± 0.170 de	61.912 ± 4.641 f	0.434 ± 0.034 d
*B. cereus* GUCC10	3.428 ± 0.148 bcd	15.000 ± 2.517 a	13.089 ± 0.436 a	2.662 ± 0.113 a	158.159 ± 6.726 a	0.585 ± 0.032 a
*B. cereus* GUCC2	3.568 ± 0.120 bc	2.333 ± 0.333 g	10.378 ± 0.503 cde	2.022 ± 0.215 cde	84.627 ± 8.160 de	0.472 ± 0.031 cd
*B. subtilis* GUCC4	3.823 ± 0.122 ab	16.500 ± 1.258 ab	12.067 ± 0.269 ab	2.539 ± 0.205 ab	112.447 ± 9.084 bc	0.549 ± 0.026 abc
*B. cereus* GUCC3	3.613 ± 0.183 bc	12.500 ± 2.466 de	12.122 ± 0.271 ab	2.390 ± 0.156 abc	116.924 ± 7.821 b	0.541 ± 0.021 abc
*B. subtilis* GUCC8	3.690 ± 0.206 bc	6.333 ± 1.856 fg	10.967 ± 0.412 cd	2.091 ± 0.163 bcd	98.676 ± 8.282 bcd	0.494 ± 0.020 bcd
*B. cereus* GUCC6	3.256 ± 0.217 cd	5.667 ± 0.882 fg	9.600 ± 0.463 e	1.568 ± 0.157 e	74.100 ± 7.419 ef	0.479 ± 0.027 cd
*B. cereus* GUCC9	3.498 ± 0.119 bc	16.667 ± 3.179 cd	11.500 ± 0.293 bc	1.933 ± 0.059 cde	95.864 ± 3.001 cd	0.490 ± 0.015 bcd

Note: Data are presented as the means ± standard deviation. Different letters in the same column indicate statistical significance (*p* < 0.05).

**Table 4:** Biological control of *B. subtilis* GUCC4 strains against leaf blight caused by *N. sphaerica* of passion fruit under greenhouse conditions.

Treatment	Disease Incidence (%)	Protection (%)	Log-Rank Test
Inoculated control	75.45	/	/
*B. subtilis* GUCC4	42.76	43.33	0.000 ***
Mancozeb	37.39	50.44	0.000 ***
*B. subtilis* NCD_2	52.72	30.12	0.002 **

Note: /: No value. **/*** indicate significant differences.

## Data Availability

The datasets presented in this study can be found in online repositories. The names of the repository/repositories and accession number(s) can be found in the article.
